# Using remote sensing environmental data to forecast malaria incidence at a rural district hospital in Western Kenya

**DOI:** 10.1038/s41598-017-02560-z

**Published:** 2017-06-01

**Authors:** Maquins Odhiambo Sewe, Yesim Tozan, Clas Ahlm, Joacim Rocklöv

**Affiliations:** 1 Kenya Medical Research Institute, Centre for Global Health Research, Box 1578, Kisumu, 40100 Kenya; 2 Umeå University, Department of Public Health and Clinical Medicine,Epidemiology and Global Health Unit, Umeå Centre for Global Health Research, Umeå, SE-901 85 Sweden; 3 New York University, College of Global Public Health, New York, 41 East 11th street, New York, NY 10003 United States; 4 Division of Social Science, New York University Abu Dhabi, Abu Dhabi, United Arab Emirates; 5 Umeå University, Department of Clinical Microbiology, Infectious Diseases, Umeå, SE-901 85 Sweden; 6 Institute of Public Health, University of Heidelberg, Im Neuenheimer Feld 324, 69120 Heidelberg, Germany

## Abstract

Malaria surveillance data provide opportunity to develop forecasting models. Seasonal variability in environmental factors correlate with malaria transmission, thus the identification of transmission patterns is useful in developing prediction models. However, with changing seasonal transmission patterns, either due to interventions or shifting weather seasons, traditional modelling approaches may not yield adequate predictive skill. Two statistical models,a general additive model (GAM) and GAMBOOST model with boosted regression were contrasted by assessing their predictive accuracy in forecasting malaria admissions at lead times of one to three months. Monthly admission data for children under five years with confirmed malaria at the Siaya district hospital in Western Kenya for the period 2003 to 2013 were used together with satellite derived data on rainfall, average temperature and evapotranspiration(ET). There was a total of 8,476 confirmed malaria admissions. The peak of malaria season changed and malaria admissions reduced overtime. The GAMBOOST model at 1-month lead time had the highest predictive skill during both the training and test periods and thus can be utilized in a malaria early warning system.

## Introduction

The year 2015 marked the end of the Millennium Development Goals and the ushering in of the new Sustainable Development Goals with continued focus on malaria as a major public health concern. By the end of 2015, the malaria incidence rate fell by 37% and the mortality rate by 60% globally^1^. Seventy percent of the reduction in malaria cases was attributed to the use of malaria prevention strategies^1^. Despite this achievement, there were still 214 million cases (range: 149–303 million) and 438,000 deaths (range: 236,000–635,000) in 2015, with 80% of the deaths concentrated in 15 countries, mainly in sub-Saharan Africa, including Kenya^1^. In sub-Saharan Africa, malaria accounts for 22% of all deaths in children aged 1–59 months^1^.

In response to this still high burden, the World Health Organization (WHO) developed the Global Technical Strategy for Malaria 2016–2030, which was adopted by the World Health Assembly in 2015. This new strategy requires reducing global malaria incidence and mortality rates by at least 90% by 2030^1^. One of the three pillars of this strategy is to use malaria surveillance as a core intervention in the control and elimination of malaria^1^. Routine malaria surveillance data provide an opportunity to develop malaria early warning systems (MEWS) to track malaria incidence and transmission patterns along with environmental risk factors for accurate and timely detection and effective control of outbreaks. The use of MEWS can help achieve the global malaria targets set for 2030.

In 2001, the WHO provided a framework for the development of MEWS in Africa^2^, centering on the use of vulnerability, transmission risk and early detection indicators^2^. Vulnerability indicators are, for example, immunity levels, migration, malnutrition, and HIV status while transmission risk indicators include climatic factors, such as rainfall and temperature. Rainfall and temperature have been used to develop malaria forecasting models. Early detection indicators, such as abrupt increases in malaria incidence, can be obtained from malaria morbidity data collected at health facilities, using epidemic thresholds, thus reinforcing the need for timely and complete reporting of malaria cases through health information systems.

Statistical methods have been used to develop regression models for early detection of epidemics of vector-borne diseases, such as malaria and dengue. For example, in endemic regions of Zambia, it was possible to detect outbreaks of malaria, by using the upper 95^th^ percentile of cases as a threshold^3^. In Singapore, models with autoregressive terms were developed for the forecasting of dengue outbreaks with a four month lead time, achieving a very high prediction accuracy^4^, while posterior predictive distributions were successfully used to classify dengue epidemic risk in Brazil^5^. In Botswana and Kenya, seasonal weather forecasts from multiple ensemble models were used to develop a MEWS with lead times up to four months^6,7^. Similar use of multiple ensemble models led to high forecast skill with a sensitivity of over 70% for seasonal forecasting of malaria incidence in India^8^. Machine learning techniques have also been used to develop malaria forecast models with high predictive skill, for example, in India^9^. Spatial temporal methods employing Bayesian statistics were employed to predict malaria transmission indicators, such as entomological inoculation rates, in Kenya^10^ and Burkina Faso^11^. Various statistical methods that have been developed and used to forecast malaria have been summarized by Zinszer *et al*.^12^.

Remote sensing provides an opportunity for spatially and temporally refined environmental data to be utilized for predictions and forecasts, especially in resource poor settings where systematic collection of temperature and rainfall data is a major challenge. It has been suggested that the development of statistical forecasting models that identify cyclic variation in malaria transmission is key to the development of MEWS for endemic regions^13^. The use of remote sensing data has been shown to improve model predictions in malaria epidemic models in the Ethiopian highlands^14^ and also in Uganda when used together with clinical predictors such as proportion screened for malaria and drug treatment^15^. A recent analysis on the effect of remote sensing data, land surface temperature (LST) and Normalized Difference Vegetation Index (NDVI) on malaria mortality showed a lagged relationship indicating an ability of forecasting based on observed data^16^.

Malaria transmission is endemic in Western Kenya, and this region suffers from high malaria morbidity and mortality. The Health and Demographic Surveillance System (HDSS) field site located in this region and run by the Kenya Medical Research Institute in collaboration with the United States Center for Disease Control (KEMRI/CDC) has the highest mortality rates compared to other HDSS field sites in the INDEPTH Network^17^, and malaria is the leading cause of death among young children under five years of age^18^. Previous studies in the KEMRI/CDC HDSS site identified patterns of lagged weather effects with malaria morbidity and mortality^16,19,20^. These studies provided potential lead times for the development of a malaria forecast model.

A malaria prediction model was previously developed for epidemic regions in Kenya, such as Wajir and Kericho, using remote sensing data^13^. A similar malaria prediction model for outbreak detection was developed and validated for the wider East African region and shown to be robust with high sensitivity and specificity^21^. This study uses remote sensing data and longitudinal malaria morbidity data from a district hospital in Western Kenya to develop and compare statistical models so as to forecast malaria admissions and assess the accuracy of these models at lead times from one to three months. Specifically, we will compare the performance of boosted and non boosted general additive models.

## Results

There was a total of 8,476 confirmed malaria admissions among children under five years of age at the Siaya district hospital during the period 2003 and 2013. Table 1 shows the summary statistics for malaria admissions by year and overall. The earlier years in the study period registered the highest number of annual admissions, with the year 2004 being the highest during which some months recorded as high as 202 pediatric malaria admissions. After 2004, the number of admissions declined gradually, but then increased to 1,249 in 2008, which was similar to what was observed in the earlier years. There was a significant drop in malaria admissions from 749 in 2009 to 166 in 2013, corresponding to a 70% reduction.

**Table 1:** Distribution of pediatric malaria admissions by year and overall at Siaya District hospital, Karemo division, Siaya county, Western Kenya, 2003–2013.

Year	Malaria admissions/year	Monthly malaria admissions Mean Min Max			Percentiles 25^th^ 50^th^ 75^th^		
		Mean	Min	Max	25^th^	50^th^	75^th^
2003	1, 258	104·8	46	168	72·0	95·5	133·5
2004	1, 468	122·3	58	202	82·0	105·0	164·0
2005	832	69·3	20	142	46·0	61·0	89·0
2006	624	52·0	22	97	32·0	41·5	71·3
2007	852	71·0	41	105	56·8	65·0	90·0
2008	1, 249	104·1	62	174	79·8	101·0	121·0
2009	749	62·4	31	78	59·5	63·5	67·5
2010	656	54·7	37	81	42·8	52·0	64·0
2011	425	35·4	11	70	26·5	29·5	42·3
2012	197	16·4	4	27	12·5	17·5	20·8
2013	166	13·8	3	27	10·0	12·5	16·5
2003–2013	8, 476	64·2	3	202	27·0	62·0	87·0

**Figure 1:**
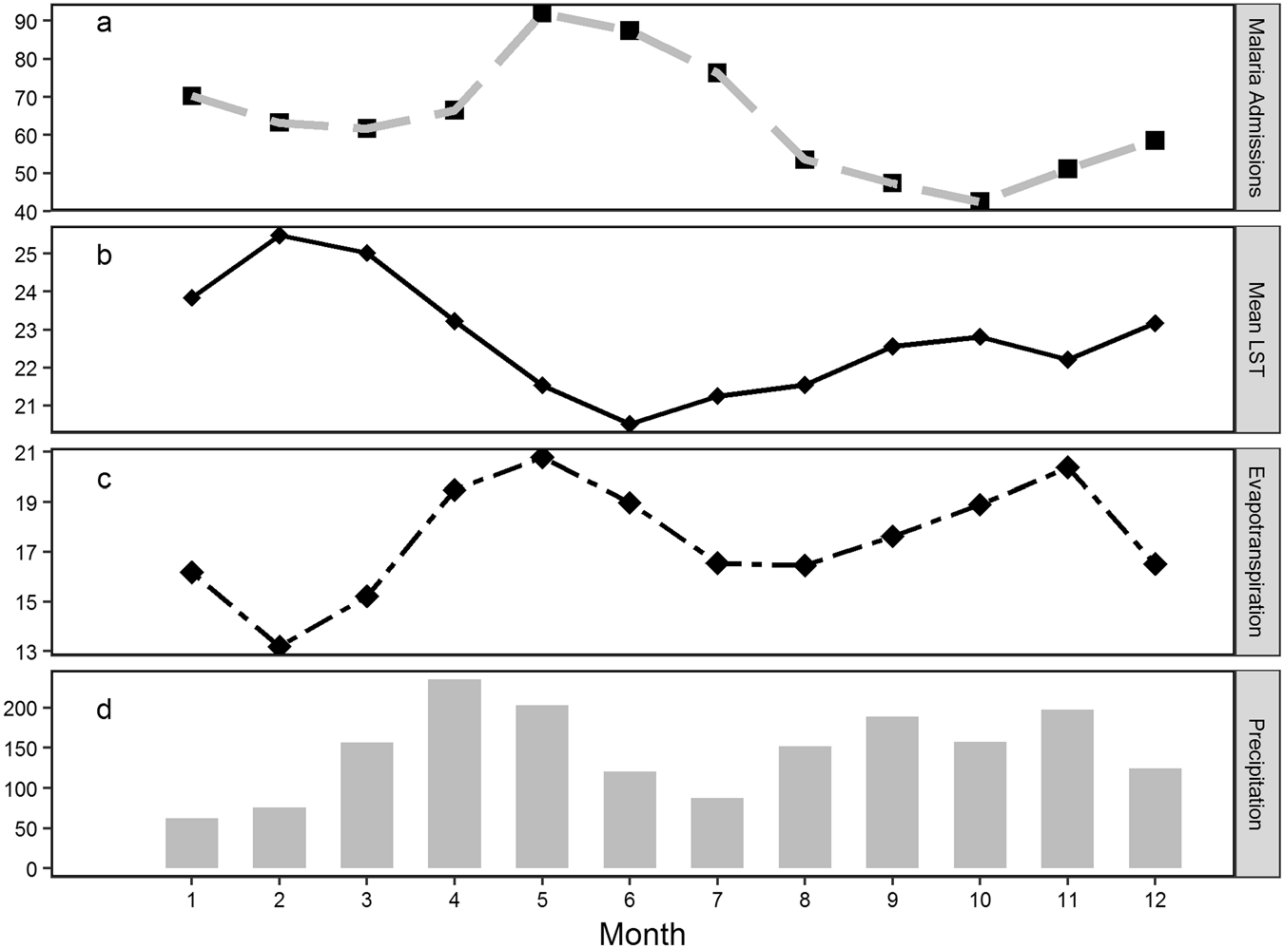
Monthly average of pediatric malaria admissions (**a**), mean LST (**b**), ET (**c**) and precipitation (**d**) in Karemo division, Siaya county, Western Kenya, 2003–2013.

Figure 1 presents mean monthly malaria admissions (Fig. 1a) and mean LST (Fig. 1b), ET (Fig. 1c) and precipitation (Fig. 1d) for the entire study period 2003–2013. The peak malaria admission months are May and June while the lowest admission month is October. The hottest month is February, while the coolest is June. The ET panel (Fig. 1c) shows that May and November are the months with the highest ET while February is the month with the least evapotranspiration. We observe two rainy seasons with the first wet months beginning in March and peaking in April, and the short rains occur from September to November. The driest months are between December and February. There is a clear lag pattern of rainfall and temperature on observed malaria admissions. From seasonal pattern, ET has the shortest lag with malaria admissions and peaks in the same month.

For precipitation, we observe a two-month lag with a peak of rainfall in April, followed by a peak in malaria admissions between May and June. For temperature, there is a longer lag of three months with a peak in February, followed again by a peak in malaria admissions between May and June.

**Figure 2:**
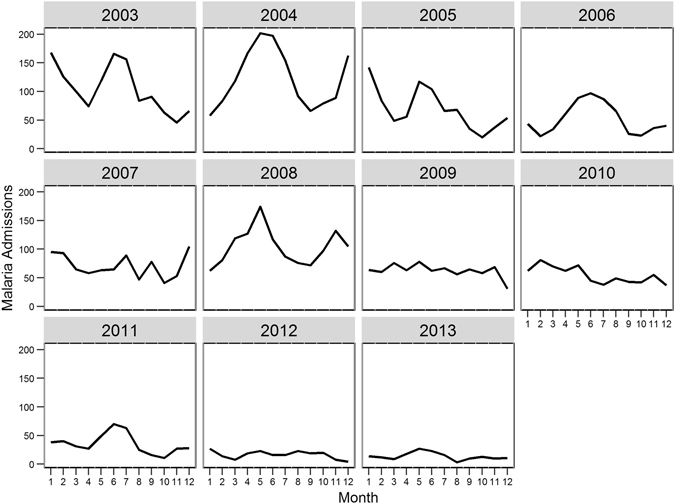
Monthly distribution of pediatric malaria admissions by year at Siaya district hospital in Karemo division, Siaya county, Western Kenya, 2003–2013.

Monthly patterns of malaria differ and the seasonal admission patterns vary across years during the study period (Fig. 2). For instance, in 2003, the admissions peaked in June and were at their lowest in November whereas in 2004, the peak was in May and the lowest admission recorded in September. We did not observe a clear seasonal pattern for the years 2007, 2009, 2010 and 2012.

### Malaria prediction models

**Figure 3:**
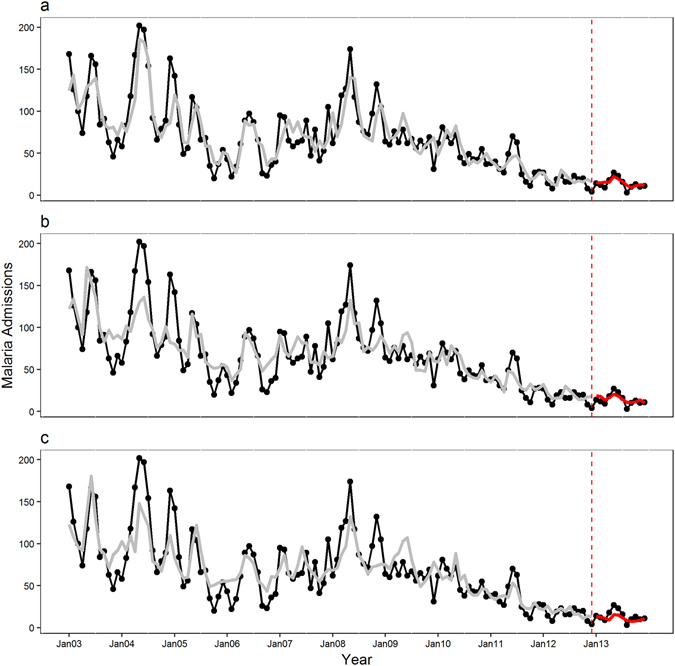
Observed and predicted pediatric malaria admissions at Siaya district hospital, Western Kenya by prediction lead time for the period 2003–2013 from the GAMBOOST model. (**a**) The 1-month, (**b**) the 2-month and (**c**) the 3-month prediction lead times respectively. The black line displays observed malaria admissions, the grey line predicted values during the training period 2003–2012, and the red line the 2013 forecasted values. The dotted red line marks the beginning of the test period.

**Figure 4:**
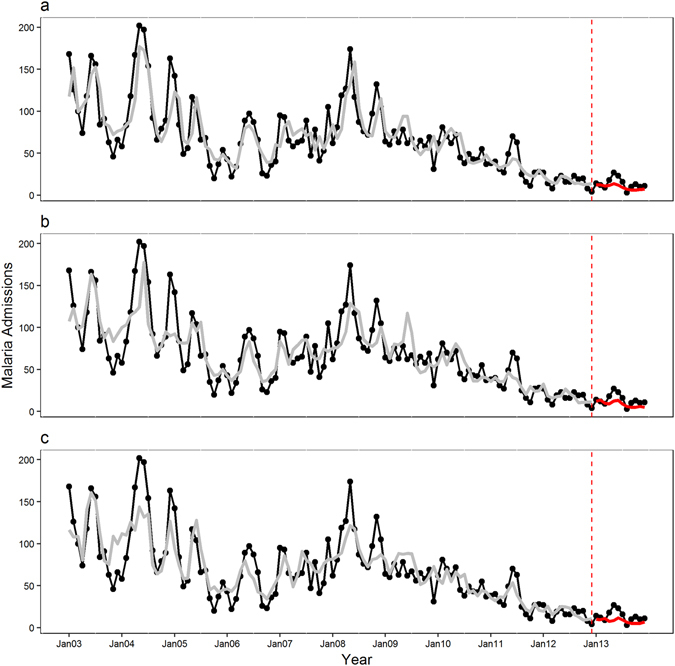
Observed and predicted pediatric malaria admissions at Siaya district hospital, Western Kenya by prediction lead time for the period 2003–2013 from the GAM model. (**a**) The 1-month, (**b**) the 2-month and (**c**) the 3-month prediction lead times respectively. The black line displays observed malaria admissions, the grey line predicted values during the training period 2003–2012, and the red line the 2013 forecasted values. The dotted red line marks the beginning of the test period.

The 1-month lead GAMBOOST model captures very well the seasonal variation in both training and test periods as displayed in Fig. 3a. It captures closely the peak malaria admissions in 2004 whereas the 2-month (Fig. 3b) and 3-month lead (Fig. 3c) models underestimate this peak. Compared to the GAMBOOST model, the 1-month lead GAM model (Fig. 4a) could not generalize well in the external data, in this case the year 2013. The generalizability of the GAM models did not improve with increasing lead times (Fig. 4b for 2-month and Fig. 4c for 3-month lead time respectively).

Supplementary Fig. S1 shows the complete external predictions for the test year of 2013 in detail for each model and lead time. Again 1-month lead models forecast closely the peak admission for the year 2013 while the 3-month model captures the peak well but underestimates the number of admissions. All the lead time GAMBOOST models overestimate the admissions in August 2013. The GAM models underestimate the malaria admissions in 2013 with only the 1-month lead model capturing the peak in the month of May correctly. The GAM models for the training period capture well the overall seasonal pattern of malaria admissions.

Table 2 displays the forecast accuracy statistics for the GAMBOOST and GAM models by lead time for the training and test periods.

**Table 2:** Forecast of pediatric malaria admissions at Siaya district hospital, Western Kenya for different prediction lead times by training and test sets including accuracy statistics.

	GAMBOOST		GAM	
**Accuracy measure**				
1-Month Lead	Training (2003–2012)	Test (2013)	Training (2003–2012)	Test (2013)
R^2^	0·80	0·71	0·77	0·44
MAE	14·53	2·98	15·33	5·26
RMSE	19·09	3·87	20·06	6·38
NMSE	0·06	0·07	0·06	0·18
NMAE	0·21	0·22	0·22	0·38
2-Month Lead				
R^2^	0·71	0·56	0·72	0·37
MAE	16·69	3·74	16·18	5·86
RMSE	22·81	4·38	22·33	6·99
NMSE	0·08	0·08	0·08	0·21
NMAE	0·24	0·27	0·24	0·42
**3-Month Lead**				
R^2^	0·73	0·50	0·74	0·16
MAE	16·50	4·19	15·77	6·70
RMSE	22·31	5·50	21·45	8·12
NMSE	0·08	0·13	0·07	0·29
NMAE	0·24	0·30	0·23	0·48

The 1-month lead GAMBOOST model explained 80% of the variation in data for the training period and 71% in the test period showing no overfitting during the training period, whereas the GAM model for the 1-month lead time explained 77% of the variance in the training set but a lower variance of 44% explained in the test dataset. Similarly, the 1-month GAMBOOST model had the lowest RMSE of 3.87 in the test period compared to 6.38 for the GAM model. In the completely external validation run, the 1-month GAMBOOST model underestimated malaria admissions by an average of 2.98 as shown with the MAE value compared to 5.26 admissions for the GAM model.

The GAMBOOST models with 2-month and 3-month lead times showed better predictions for the test period and also had better predictive accuracy compared with the GAM models, with the GAM model with 3-month lead time showing the worst prediction accuracy with an R^2^ of 16% compared to the training period of 74% showing overfitting, as to be compared to the GAMBOOST model for the same time showing an R^2^ of 50% and 73%, respectively.

## Discussion

To forecast monthly pediatric malaria admissions at a district hospital in Western Kenya, we developed two structurally different models using satellite data of LST, ET and precipitation with a lead time of 1 to 3 months. We utilized a robust validation scheme of 5-fold cross-validation and withheld the year 2013 from the model building to infer the model’s predictive generalizability. We found one of the model structures involving generalized additive models with a boosting algorithm providing the best forecasts at all lead times.

The basic reproduction number (R_0_) for malaria depends on a number of factors, such as mosquito biting rate, mosquito density and extrinsic incubation period of malaria parasites in the mosquito host. All of these factors are affected by temperature^22,23^ and rainfall^24,25^. At suitable temperatures, mosquito development time is reduced thus providing stable transmission in endemic regions, such as Western Kenya. We used satellite derived LST, precipitation and ET as proxies to these factors at various forecast lead times. The lead time of forecast provides a window for users of the forecast information, such as malaria control managers, to act.

The seasonal distribution of malaria admissions in the study area changed considerably and exhibited a decreasing trend over time with an abrupt increase observed in 2008. Similar patterns have been observed in other areas in Western Kenya between 2002 and 2010^26^. This could be due to several factors, including interventions, sudden movements of susceptible people into endemic areas (e.g. the migration of people back into the study area after the post-election violence in Kenya in 2008^27^), and changes in the seasonality of environmental conditions due to climate variability and El Nino years^28^. The varying annual peak in admissions is a challenge for developing forecasting models in endemic settings that rely on cyclic pattern of disease transmission.

Our analysis has shown that boosting regression methods can help improve model fit through iterative variable selection. This makes the regression parameters chosen to be stable even if the mean trend of malaria incidence changes with the use of control strategies. The GAMBOOST method has been shown to better fit data that is non stationary^29^, as the variance of the response variable can be time dependent in this model. In all the models with different lead times, the GAMBOOST models captured well the variation during the training and testing of data. This indicated that the model greatly reduced overfitting, resulting in better forecast accuracy. The normalized accuracy parameters were very comparable between the 5-fold cross-validation and the 2013 test period. In comparison, the GAM model optimized the coefficients for the training period but could not capture the patterns well in the out of sample 2013 dataset resulting in poor predictions in most of the out of sample test series. The GAM model could not identify correctly the peak months of malaria admissions and underestimated the number of admissions. This means that the model over fitted the training data and thus had very unstable or biased regression parameters.

Early warning systems rely on thresholds to issue alerts. Models that under-predict are likely to fail in issuing warnings when there are true epidemics while models that over-predict can potentially issue false alerts. The GAMBOOST models had the least mean absolute errors in the validation period, which suggested that they could potentially be used to issue alerts based on thresholds. Depending on the thresholds set, the GAMBOOST model can potentially underestimate high transmission months. However, this malaria endemic setting has no set threshold. The prediction on increase in malaria admissions can trigger response action without necessarily considering the magnitude in this situation. Malaria control managers could define a threshold for more simple control response routines. The prediction accuracy of outbreak/no outbreak could then be estimated using reciever operating characteristic curves and the area under the curve (AUC), and such methods allow for tuning of the outbreak probability threshold. Thus, even a lower prediction, which picks up correct outbreak pattern, would yield high sensitivity and specificity by the AUC after the calibration to the set threshold.

The GAMBOOST and GAM models provided better prediction at a lag of one month. This is mainly because the number of malaria cases in a particular month is strongly correlated with the number of cases in the preceding month than those two or three months before. This is consistent with most models using autoregressive terms for monthly malaria forecasts^9,20^. A model with two to four months lead time was developed for epidemic prediction in the Western Kenyan highlands^30^. The one-month lead time is very short for action. However, given that this is an endemic area, intervention strategies can potentially be marshalled in a short period if epidemic preparedness and response strategies are in place. Similarly, actions can be fine-tuned or intensified when lead time and uncertainty decrease with models consistently identifying epidemic patterns. The model can be improved to provide longer lead times by using seasonal forecasts, which provide lead times ranging from one to six months^6^.

This study has a number of limitations. The time series data used covered periods during which a number of vector control strategies were implemented in Karemo division in Siaya county. Indoor residual spraying began in 2004 in Karemo, and insecticide treated bednet use was scaled up 2006 onwards^31,32^. Because of malaria interventions, malaria incidence does not correlate well with seasonal weather forecasts, and it has been suggested that data collected during malaria control periods should not be used for model training^21^. The interventions implemented in the study area over the study period might have had an impact on the inter-annual variation in malaria transmission and the long-term trends. To improve prediction accuracy, it is important to account for these intervention measures in the models. The main challenge is to determine when an intervention started, how long it was implemented and what the extent of its coverage was to correctly include it in the time series data. Therefore, we suggest further time series analyses to identify intervention periods and intensity levels. Several unmeasured factors in this study could have acted non-linearly to affect malaria transmission. In this study, it was impossible to consider all these factors in the model. To account for these unmeasured factors, we used spline of the trend function, which may not be sufficient to capture all the complex processes affecting malaria transmission. In this analysis, the satellite data was aggregated over a large area and thus reduced spatial accuracy. By use of high resolution data, it would be possible to develop high-resolution spatial-temporal models to capture malaria transmission and attain better predictive accuracy.

The models developed in this study were purely for prediction purposes; therefore, we chose only models with high prediction accuracy. Consequently, we cannot infer the effect of remote sensing factors on malaria morbidity. Another limitation of this study is measurement errors on environmental data, as well as malaria incidence data. The limitation due to the quality of satellite data can be circumvented by integrating locally collected environmental data. For example, the predictive accuracy of the model can be improved by using datasets that combine both satellite and ground data, for example the climate data that will become available from the Enhancing National Climate Services initiative (ENACTS)^33^.

Different regions have varying malaria epidemiology; therefore, the model should be tested and validated before its deployment to other areas. Lastly, we used same lag times for all environmental variables in the model. As evident from other studies, the lagged patterns with malaria indicators, however, vary for each term^16,19^.

In conclusion, two different models using satellite data for LST, precipitation and ET were tested to forecast pediatric malaria admissions in Western Kenya. The GAMBOOST model with a lead time of 1 month proved to have the best accuracy to predict monthly admissions at a district hospital. This lead time may be short but can provide enough time to intensify malaria control interventions in an endemic area where a malaria preparedness and response plan is in place.

This study shows that the use of boosting regression in GAM models can be beneficial in early warning systems to improve predictions. We hope that our findings would encourage the continued use of GAMBOOST in early warnings systems and the wider development and use of early warnings in malaria control.

## Methods

### Study setting and malaria data

The study is based at the KEMRI/CDC HDSS field site in Western Kenya. The KEMRI/CDC HDSS has been operational in Asembo since 2001. It expanded to include Gem in 2002 and Karemo in 2007. The HDSS monitors the health and demographic changes in the study population through routine collection of health data at health care facilities and demographic and socio-economic data from households. Over 240,000 individuals are under surveillance. Some of the demographic information monitored include births, deaths, and migration. Information on cause of death is also collected through verbal autopsy. Morbidity data have been routinely collected at the health facilities in the HDSS area. Hospital-based surveillance is currently conducted at three health facilities; inpatient data are routinely collected at the Siaya district hospital, and outpatient data at the health facilities in Njenjra and Ting Wang’i. The Siaya district hospital is a referral hospital in Karemo division of Siaya county. The KEMRI/CDC HDSS has been described in detail elsewhere^34,35^.

In this study, we used malaria admissions data collected at the Siaya district hospital for the period 2003–2013. The hospital surveillance data were complete for this period and collected routinely by the health care workers employed by the KEMRI/CDC. We extracted the admissions data for children under five years of age with confirmed *Plasmodium falciparum* malaria. The data were then aggregated to monthly time scale for each year to create a time series dataset.

### Satellite environmental data

We used satellite derived day and night LSTs, NDVI and precipitation data for the period 2003–2013. Rainfall estimates were extracted from NASA’s Tropical Rainfall Measuring Mission (TRMM) 3B42_V7 Product for daily accumulated rainfall available at 0.25° by 0.25° spatial resolution. Day and night LSTs were extracted from the Moderate Resolution Imaging Spectro-radiometer (MODIS) MOD11A1 product with a 1-kilometer spatial resolution and daily temporal resolution. We took an average of the day and night LSTs to get a mean LST. In addition to these variables, we also included evapotranspiration data from the MODIS product MOD16 available at 8 days temporal and 1-kilometer spatial resolution. The detailed processing of these datasets were described in an earlier study^16^. These datasets were aggregated to monthly summaries. We computed monthly totals for rainfall and monthly averages for the other environmental factors.

### Statistical analysis

We used a general additive modelling framework to build forecast models for malaria admissions, with smooth functions of environmental factors at different lead times. Studies have shown nonlinear relationships between weather factors and malaria morbidity and mortality^16,19,36–39^. We developed two different general additive models, one using a boosting algorithm to optimize model fit and the other without boosting.

The malaria admissions data used in this study exhibited over-dispersion. In a Poisson distribution, the mean and variance are equal. Over-dispersion occurs when variance is greater than the mean. To account for over-dispersion, we assumed negative binomial distribution in both models.

### General Additive Model (GAM)

The general additive model (GAM) without boosting was developed using the *mgcv* package in R^40^. The model included a cubic regression spline of time to adjust for the overall trend in malaria admissions during the study period. To address the observed within-year seasonality of malaria, we used a cyclic cubic regression function of month to capture the peaks in malaria admissions. Mean LST, ET and precipitation were included as cubic regression splines in the model.

Malaria cases in any given month are likely to be correlated with malaria cases in preceding months. The number of previously infected individuals determines the reservoir of infectious mosquitoes, which in turn affects the current population of infected individuals. To control for this autocorrelation, we included previous malaria cases as autoregressive terms (AR) in the models for each lead time. Previous studies in this HDSS area^20^ and in Burundi^41^ included a 1-month AR term to adjust for autocorrelation. We also included a simple random effect spline function of month. Smoothing degrees of freedom were optimally determined using general cross validation.

To assess different prediction lead times, three separate models were developed with 1-month, 2-month and 3-month lead times. To attain a 1-month lead time we took a lag of one month of environmental factors and malaria cases and for the 2-month and 3-month lead times we took a lag of two and three months, respectively.

The model equations were:

1 $$\begin{array}{ccc}{\rm{l}}{\rm{o}}{\rm{g}}({y}_{t}) & = & {\rm{s}}({\rm{t}}{\rm{i}}{\rm{m}}{\rm{e}})+{\rm{s}}({\rm{m}}{\rm{o}}{\rm{n}}{\rm{t}}{\rm{h}},{\rm{b}}{\rm{s}}={\mbox{''}}{\rm{c}}{\rm{c}}\mbox{''})+{\rm{s}}({{\rm{L}}{\rm{S}}{\rm{T}}}_{t-1})+{\rm{s}}({{\rm{P}}{\rm{r}}{\rm{e}}{\rm{c}}{\rm{i}}{\rm{p}}{\rm{i}}{\rm{t}}{\rm{a}}{\rm{t}}{\rm{i}}{\rm{o}}{\rm{n}}}_{t-1})+{\rm{s}}({{\rm{E}}{\rm{T}}}_{t-1})\\  &  & +{\rm{s}}({\rm{m}}{\rm{o}}{\rm{n}}{\rm{t}}{\rm{h}},{\rm{b}}{\rm{s}}={\mbox{''}}{\rm{r}}{\rm{e}}{\textstyle \text{''}})+{\rm{s}}({{\rm{M}}{\rm{A}}{\rm{L}}}_{t-1})\end{array}$$

2 $$\begin{array}{ccc}{\rm{l}}{\rm{o}}{\rm{g}}({y}_{t}) & = & {\rm{s}}({\rm{t}}{\rm{i}}{\rm{m}}{\rm{e}})+{\rm{s}}({\rm{m}}{\rm{o}}{\rm{n}}{\rm{t}}{\rm{h}},{\rm{b}}{\rm{s}}={\mbox{''}}{\rm{c}}{\rm{c}}\mbox{''})+{\rm{s}}({{\rm{L}}{\rm{S}}{\rm{T}}}_{t-2})+{\rm{s}}({{\rm{P}}{\rm{r}}{\rm{e}}{\rm{c}}{\rm{i}}{\rm{p}}{\rm{i}}{\rm{t}}{\rm{a}}{\rm{t}}{\rm{i}}{\rm{o}}{\rm{n}}}_{t-2})+{\rm{s}}({{\rm{E}}{\rm{T}}}_{t-2})\\  &  & +{\rm{s}}({\rm{m}}{\rm{o}}{\rm{n}}{\rm{t}}{\rm{h}},{\rm{b}}{\rm{s}}={\mbox{''}}{\rm{r}}{\rm{e}}{\textstyle \text{''}})+{\rm{s}}({{\rm{M}}{\rm{A}}{\rm{L}}}_{t-2})\end{array}$$

3 $$\begin{array}{ccc}{\rm{l}}{\rm{o}}{\rm{g}}({y}_{t}) & = & {\rm{s}}({\rm{t}}{\rm{i}}{\rm{m}}{\rm{e}})+{\rm{s}}({\rm{m}}{\rm{o}}{\rm{n}}{\rm{t}}{\rm{h}},{\rm{b}}{\rm{s}}={\mbox{''}}{\rm{c}}{\rm{c}}\mbox{''})+{\rm{s}}({{\rm{L}}{\rm{S}}{\rm{T}}}_{t-3})+{\rm{s}}({{\rm{P}}{\rm{r}}{\rm{e}}{\rm{c}}{\rm{i}}{\rm{p}}{\rm{i}}{\rm{t}}{\rm{a}}{\rm{t}}{\rm{i}}{\rm{o}}{\rm{n}}}_{t-3})+{\rm{s}}({{\rm{E}}{\rm{T}}}_{t-3})\\  &  & +{\rm{s}}({\rm{m}}{\rm{o}}{\rm{n}}{\rm{t}}{\rm{h}},{\rm{b}}{\rm{s}}={\mbox{''}}{\rm{r}}{\rm{e}}{\textstyle \text{''}})+{\rm{s}}({{\rm{M}}{\rm{A}}{\rm{L}}}_{t-3})\end{array}$$

*Y*_*t*_ ~Negative Binomial

where s is a smoothing spline; bs = “cc” is the cyclic cubic regression spline basis function of month to control for seasonality; bs = “re” is the random effect spline basis; and MAL represents the autoregressive malaria cases. The other spline functions are cubic regression splines. Models (1), (2), and (3) correspond to 1-month, 2-month and 3-month prediction lead times, respectively.

### General Additive Model with boosting (GAMBOOST)

The general additive model with boosting was developed using *gamBoostlss*^42,43^ package in R. The *gamBoostlss* is a regression boosting method for GAMs encompassing location, scale and shape. The method uses a gradient boosting algorithm for variable smoothing selection. The model starts with weak base learners and in each iteration optimizes the model. In each subsequent iteration, only variables selected up to the current iteration are included. Similar to the GAM model, we used smooth base learners of time, Mean LST, ET, precipitation and previous malaria cases as AR terms for each lead time. We also include a random base learner for month and a cyclic base learner for month. The equations for each model are as follows:

4 $$\begin{array}{ccc}{\rm{l}}{\rm{o}}{\rm{g}}({y}_{t}) & = & {\rm{b}}{\rm{b}}{\rm{s}}({\rm{t}}{\rm{i}}{\rm{m}}{\rm{e}})+{\rm{b}}{\rm{b}}{\rm{s}}({\rm{m}}{\rm{o}}{\rm{n}}{\rm{t}}{\rm{h}},{\rm{c}}{\rm{y}}{\rm{c}}{\rm{l}}{\rm{i}}{\rm{c}}={\rm{T}})+{\rm{b}}{\rm{b}}{\rm{s}}({{\rm{L}}{\rm{S}}{\rm{T}}}_{t-1})+{\rm{b}}{\rm{b}}{\rm{s}}({{\rm{P}}{\rm{r}}{\rm{e}}{\rm{c}}{\rm{i}}{\rm{p}}{\rm{i}}{\rm{t}}{\rm{a}}{\rm{t}}{\rm{i}}{\rm{o}}{\rm{n}}}_{t-1})\\  &  & +{\rm{b}}{\rm{b}}{\rm{s}}({{\rm{E}}{\rm{T}}}_{t-1})+{\rm{b}}{\rm{r}}{\rm{a}}{\rm{n}}{\rm{d}}{\rm{o}}{\rm{m}}({\rm{m}}{\rm{o}}{\rm{n}}{\rm{t}}{\rm{h}})+{\rm{b}}{\rm{b}}{\rm{s}}({{\rm{M}}{\rm{A}}{\rm{L}}}_{t-1})\end{array}$$

5 $$\begin{array}{ccc}{\rm{l}}{\rm{o}}{\rm{g}}({y}_{t}) & = & {\rm{b}}{\rm{b}}{\rm{s}}({\rm{t}}{\rm{i}}{\rm{m}}{\rm{e}})+{\rm{b}}{\rm{b}}{\rm{s}}({\rm{m}}{\rm{o}}{\rm{n}}{\rm{t}}{\rm{h}},{\rm{c}}{\rm{y}}{\rm{c}}{\rm{l}}{\rm{i}}{\rm{c}}={\rm{T}})+{\rm{b}}{\rm{b}}{\rm{s}}({{\rm{L}}{\rm{S}}{\rm{T}}}_{t-2})+{\rm{b}}{\rm{b}}{\rm{s}}({{\rm{P}}{\rm{r}}{\rm{e}}{\rm{c}}{\rm{i}}{\rm{p}}{\rm{i}}{\rm{t}}{\rm{a}}{\rm{t}}{\rm{i}}{\rm{o}}{\rm{n}}}_{t-2})\\  &  & +{\rm{b}}{\rm{b}}{\rm{s}}({{\rm{E}}{\rm{T}}}_{t-2})+{\rm{b}}{\rm{r}}{\rm{a}}{\rm{n}}{\rm{d}}{\rm{o}}{\rm{m}}({\rm{m}}{\rm{o}}{\rm{n}}{\rm{t}}{\rm{h}})+{\rm{b}}{\rm{b}}{\rm{s}}({{\rm{M}}{\rm{A}}{\rm{L}}}_{t-2})\end{array}$$

6 $$\begin{array}{ccc}{\rm{l}}{\rm{o}}{\rm{g}}({y}_{t}) & = & {\rm{b}}{\rm{b}}{\rm{s}}({\rm{t}}{\rm{i}}{\rm{m}}{\rm{e}})+{\rm{b}}{\rm{b}}{\rm{s}}({\rm{m}}{\rm{o}}{\rm{n}}{\rm{t}}{\rm{h}},{\rm{c}}{\rm{y}}{\rm{c}}{\rm{l}}{\rm{i}}{\rm{c}}={\rm{T}})+{\rm{b}}{\rm{b}}{\rm{s}}({{\rm{L}}{\rm{S}}{\rm{T}}}_{t-3})+{\rm{b}}{\rm{b}}{\rm{s}}({{\rm{P}}{\rm{r}}{\rm{e}}{\rm{c}}{\rm{i}}{\rm{p}}{\rm{i}}{\rm{t}}{\rm{a}}{\rm{t}}{\rm{i}}{\rm{o}}{\rm{n}}}_{t-3})\\  &  & +{\rm{b}}{\rm{b}}{\rm{s}}({{\rm{E}}{\rm{T}}}_{t-3})+{\rm{b}}{\rm{r}}{\rm{a}}{\rm{n}}{\rm{d}}{\rm{o}}{\rm{m}}({\rm{m}}{\rm{o}}{\rm{n}}{\rm{t}}{\rm{h}})+{\rm{b}}{\rm{b}}{\rm{s}}({{\rm{M}}{\rm{A}}{\rm{L}}}_{t-3})\end{array}$$

*Y*_*t*_ ~Negative Binomial

where *bbs* is the smooth base learner. The smooth base learner for month is set to be cyclic to control for seasonality. Random is the random base learner for month. MAL represents the autoregressive malaria cases. Models (4), (5), and (6) correspond to 1-month, 2-month and 3-month prediction lead times, respectively.

### Model validation

To get an optimal number of boosting iterations we performed k-fold cross validation on the training dataset. K-fold cross validation involves partitioning the training data into k subsets. In each run, one subset is held for validation while the remaining k-1 subsets are used for model fitting. The number of iterations giving the lowest prediction in the k out of sample set is chosen.

We performed 5-k fold validation with 1,000 initial iterations with 0·01 step to get the number of boosting iterations for the *gamboostlss* model. To assess the predictive ability of the models, we split the data into training and testing datasets. The time series for the period 2003–2012 was used for model training while the 2013-time series for model testing. R-squared statistic, root mean squared error (RMSE), normalized mean squared error (NMSE), mean absolute error (MAE) and normalized mean absolute error (NMAE) were used for model comparison. The equations for these measures are given below:

$$MAE=\frac{1}{n}\sum _{i=1}^{n}| {e}_{i}^{2}| $$

$$RMSE=\sqrt{\frac{1}{n}\sum _{i=1}^{n}{e}_{i}^{2}}$$

$$NMSE=\frac{1}{{\bar{Y}}}\sqrt{\frac{1}{n}\sum _{i=1}^{n}{e}_{i}^{2}}$$

where $\bar{Y}$ is the scaling factor

where *e*_*i*_ = *f*_*i*_ − *y*_*i*_, *f*_*i*_ is the prediction and *y*_*i*_, the observed value.

The NMAE is scaled using the lowest and the highest values in the series.

These measures have been explained in details in *Shcherbakov et al*.^44^. We included the normalized measures to be able to assess prediction accuracy between training and test periods. These measures are relevant when there are different scales^44^; in this case mean malaria admissions differ between test and training periods.

All analysis was done using R statistical software^45^. The *DMwR*^46^ package was used to produce the forecast accuracy statistics.

### Data availability

The datasets generated during and/or analysed during the current study are available from the corresponding author on reasonable request.

### Ethics Statement

The protocols for KEMRI/CDC HDSS are approved by both CDC (#3308, Atlanta, GA) and KEMRI (#1801, Nairobi, Kenya) Institutional Review Boards. Informed consent was obtained from all the participants. The study was ethically conducted adhering to the Helsinki declaration and current ethical guidelines.

## Electronic supplementary material

Supplementary files
