# Author Correction: Using remote sensing environmental data to forecast malaria incidence at a rural district hospital in Western Kenya

**DOI:** 10.1038/s41598-018-23241-5

**Published:** 2018-03-19

**Authors:** Maquins Odhiambo Sewe, Yesim Tozan, Clas Ahlm, Joacim Rocklöv

**Affiliations:** 1 Kenya Medical Research Institute, Centre for Global Health Research, Box 1578, Kisumu, 40100 Kenya; 2 Umeå University, Department of Public Health and Clinical Medicine,Epidemiology and Global Health Unit, Umeå Centre for Global Health Research, Umeå, SE-901 85 Sweden; 3 New York University, College of Global Public Health, New York, 41 East 11th street, New York, NY 10003 United States; 4 Division of Social Science, New York University Abu Dhabi, Abu Dhabi, United Arab Emirates; 5 Umeå University, Department of Clinical Microbiology, Infectious Diseases, Umeå, SE-901 85 Sweden; 6 Institute of Public Health, University of Heidelberg, Im Neuenheimer Feld 324, 69120 Heidelberg, Germany

Correction to: *Scientific Reports* 10.1038/s41598-017-02560-z, published online 01 June 2017

This Article contains an error in the legend of Figure 1 where the units were omitted. The correct legend appears below:

Figure 1: Monthly average of pediatric malaria admissions (**a**), mean LST (°C) (**b**), ET(mm/day) (**c**) and precipitation (mm) (**d**) in Karemo division, Siaya county, Western Kenya, 2003–2013.

