# Supplementary material for: Using remote sensing environmental data to forecast malaria incidence at a rural district hospital in Western Kenya
Source: Sci Rep. 2017 Jun 1;7:2589. doi: 10.1038/s41598-017-02560-z (PMC5453969; doi:10.1038/s41598-017-02560-z)

Using remote sensing environmental data to forecast malaria incidence at a rural district hospital in Western Kenya

Maquins Odhiambo Sewe [1, 2*], Yesim Tozan [3,5], Clas Ahlm [4], Joacim Rocklöv [2]

[1] Kenya Medical Research Institute, Centre for Global Health Research, Kisumu, Box 1578,40100, Kenya,

[2] Umeå University, Department of Public Health and Clinical Medicine, Epidemiology and Global Health, Umeå, SE-901 85, Sweden

[3] New York University, College of Global Public Health, New York,41 East 11th street, New York, NY 10003, United States

[4] Umeå University, Department of Clinical Microbiology, Infectious Diseases, Umeå, SE-90185, Sweden

[5] Heidelberg University Medical School, Institute of Public Health, Heidelberg, Im Neuenheimer Feld 324, 69120 Heidelberg, Germany

* Corresponding author, sewemaquins@gmail.com

Supplementary Table S1. Distribution of mean LST Karemo 2003-2013

				Percentiles		
Year	Mean	Min	Max	25th	50th	75th
2003	22.97	19.44	26.35	21.65	23.01	24.28
2004	23.30	20.86	25.66	22.30	23.02	24.22
2005	23.56	21.16	27.20	22.14	22.53	25.32
2006	22.92	20.26	28.37	21.03	22.03	23.74
2007	21.87	19.59	24.72	20.69	22.19	22.49
2008	22.42	20.46	25.31	21.16	21.86	23.87
2009	23.11	20.98	24.90	22.43	22.75	24.03
2010	22.29	20.58	24.27	21.50	22.21	23.06
2011	22.56	19.98	25.90	21.40	22.04	23.83
2012	22.66	17.23	27.32	21.17	22.58	24.68
2013	22.75	21.52	25.13	21.94	22.10	23.86
2003-2013	22.77	17.23	28.37	21.51	22.46	24.11

Supplementary Table S2. Distribution of precipitation Karemo 2003-2013

				Percentiles		
Year	Mean	Min	Max	25th	50th	75th
2003	139.85	65.94	242.44	105.39	121.78	163.10
2004	129.45	49.43	310.03	75.94	97.75	164.80
2005	121.75	13.45	304.48	85.30	108.48	144.28
2006	207.88	69.27	441.57	142.25	208.75	232.95
2007	137.36	83.66	197.14	115.66	136.00	161.59
2008	151.10	39.60	255.17	104.05	173.06	190.39
2009	131.05	41.36	253.35	67.03	117.37	207.76
2010	138.91	62.31	213.86	122.72	140.15	158.43
2011	156.81	41.38	372.71	59.12	160.76	189.86
2012	156.94	4.55	290.73	67.26	179.96	231.44
2013	141.54	33.01	280.83	72.07	122.86	186.33
2003-2013	146.60	4.55	441.57	86.98	136.92	193.86

Supplementary Table S3. Distribution of Evapotranspiration Karemo 2003-2013

				Percentiles		
Year	Mean	Min	Max	25th	50th	75th
2003	17.81	11.04	20.79	17.40	18.33	19.30
2004	17.18	14.30	21.08	14.96	17.09	18.91
2005	17.20	11.44	21.59	16.72	17.69	18.87
2006	18.18	11.08	22.06	17.28	18.93	20.44
2007	19.63	16.05	22.05	19.35	19.75	20.10
2008	17.80	13.26	20.97	16.28	18.07	20.02
2009	16.52	12.79	20.81	13.57	16.94	19.11
2010	17.50	13.60	21.57	16.07	17.52	18.70
2011	16.70	9.25	21.66	14.27	17.22	19.11
2012	16.96	8.81	21.49	16.27	18.39	19.67
2013	17.24	14.37	20.48	15.15	17.59	18.81
2003-2013	17.52	8.81	22.06	15.73	18.16	19.86

Supplementary Table S4. GAMBOOST and GAM model predictions for malaria admissions 2013 by lead time

		GAMBOOST			GAM		
2013		Lead Time			Lead Time		
Month	Observed	1-Month	2-Month	3-Month	1-Month	2-Month	3-Month
Jan	14	15.10	12.42	18.36	12.27	13.32	10.06
Feb	12	14.94	12.51	18.23	13.65	12.58	9.63
Mar	9	15.59	10.97	13.71	9.85	11.26	10.83
Apr	18	15.52	11.58	16.41	9.14	9.31	7.43
May	27	22.48	13.75	20.55	12.62	15.44	8.41
Jun	23	19.23	12.17	18.40	13.43	14.97	11.71
Jul	16	16.05	9.28	14.13	8.92	11.83	9.29
Aug	3	11.75	7.15	10.77	5.99	8.72	6.63
Sep	10	9.37	6.08	10.66	4.90	7.63	5.19
Oct	13	11.40	6.29	11.39	4.96	7.83	5.07
Nov	10	12.25	6.80	14.09	5.86	8.95	5.26
Dec	11	12.10	7.14	10.10	5.02	11.03	7.00

Supplementary Figure S1. GAMBOOST (a,b,c for 1-month, 2-month and 3-month prediction lead times) and GAM (d,e,f for 1-month, 2-month and 3-month prediction lead times) predictions for malaria admissions 2013 by lead time. The green lines are the model predictions.

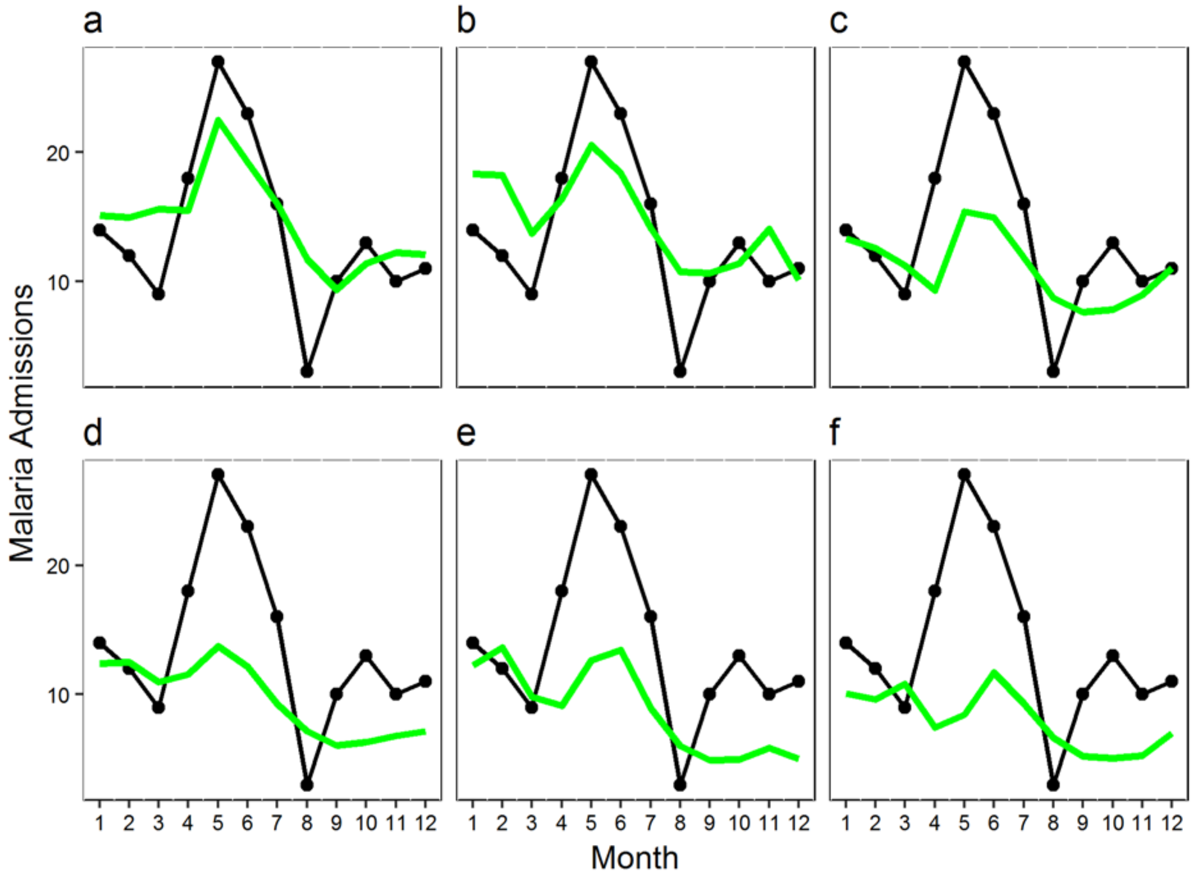

Supplement: Supplementary file 1 — Supplementary files [file 41598_2017_2560_MOESM1_ESM.pdf]
